# Comparison of microbial communities from different Jinhua ham factories

**DOI:** 10.1186/s13568-017-0334-0

**Published:** 2017-02-13

**Authors:** Qingfeng Ge, Yubin Gu, Wangang Zhang, Yongqi Yin, Hai Yu, Mangang Wu, Zhijun Wang, Guanghong Zhou

**Affiliations:** 1 Key Lab of Meat Processing and Quality Control, Jiangsu Collaborative Innovation Center of Meat Production and Processing, Quality and Safety Control, College of Food Science and Technology, Nanjing Agricultural University, Nanjing, 210095 Jiangsu China; 2 College of Food Science and Engineering, Yangzhou University, Yangzhou, 225127 Jiangsu China

**Keywords:** Jinhua ham, Pyrosequencing, Microbial communities, Flavor, Aldehydes

## Abstract

Microbes in different aged workshops play important roles in the flavor formation of Jinhua ham. However, microbial diversity, community structure and age related changes in workshops are poorly understood. The microbial community structure and diversity in Jinhua ham produced in factories that have 5, 15, and 30 years of history in processing hams were compared using the pyrosequencing technique. Results showed that 571,703 high-quality sequences were obtained and located in 242 genera belonging to 18 phyla. Bacterial diversity and microbial community structure were significantly different with the years of workshops. Three-phase model to characterize the changes of ham microbial communities was proposed. Gas chromatography–mass spectrometry assays indicated that the hams produced in different aged workshops have great differences in number and relative contents of volatiles compounds. These results suggest that different aged factories could form special and well-balanced microbial diversity, which may contribute to the unique flavor characteristics in Jinhua ham.

## Introduction

Previous studies have demonstrated that the unique and the broad diversity of flavors in ham are the result of complex reactions including lipid oxidation, Maillard reactions and protein degradation (Zhang et al. 2009; Zhou and Zhao 2007). These reactions mainly depend on enzymatic action of endogenous enzymes and microorganisms (Antequera et al. 1992; Petrova et al. 2015; Ventanas et al. 2008; Zhou and Zhao 2007). Hence, in the past two decades, the composition of microbial communities and main flora in ham has been widely investigated (Fulladosa et al. 2010; Martín et al. 2006). However, most of these researches have focused on the change of microbial diversity during the processing of ham. There are few reports concerning the differences in the microbial community structure in the ham that produced in different manufacturing places. In the ham industry, it is generously accepted that the ham produced in different workshop has its unique flavor characteristics. The quality of ham is attributed to the maturing process of workshop which has a well-balanced microbial community structure and diversity in the ham. It is thus highly interesting to investigate the microorganism community structure of dry-cured ham produced in different workshops.

Community-level studies have become more precise with the application of culture-independent methods based on the direct detection of DNA in microbial ecosystems. The pyrosequencing techniques is the effective molecular tool for describing comprehensive diversities of microflora and has been successfully applied in fresh meat products (Xiao et al. 2013; Zhao et al. 2015) and sausages (Połka et al. 2015; Rebecchi et al. 2015) to understand the changes in the microbial populations during production or storage. Hence, applying the pyrosequencing technique in dry-cured ham can give new insight and comprehensive understanding of the microbial community structure.

Jinhua ham, a representative of traditional dry-cured meat product from Zhejiang Province in Eastern China, is considered as a high quality product with unique flavor. In the present study, the flavor characteristics of Jinhua ham produced in those workshops have 5, 15, and 30 years of history in processing hams and the structure and diversity of the microbial community of these hams were investigated by gas chromatography–mass spectrometry (GC–MS) and the 16S rDNA gene pyrosequencing technique, respectively. The results were expected to provide insights into the microbial community structure and diversity among different aged workshops which produced special flavor Jinhua hams.

## Materials and methods

### Processing of Jinhua ham and sampling

Jinhua hams were processed in three workshops following the same traditional technology with same batch of green hams in Zhejiang Provincial Food Company, PR China. The traditional process divided into six phases: natural cooling, salting, soaking and washing, sun-drying, loft-aging and post-aging (Huan et al. 2005). According to the history of these three workshops, they were distinguished as JN (30 years), JD (15 years) and JM (5 years). All of these three workshops located in the Jindong district of Jinhua City, Zhejiang Province, their coordinates are 119° 84′E-119° 98′ E and 29° 28′ N-29° 46′ N.

At the middle of aging, hams were taken as samples for DNA extraction and volatile compounds analysis. Three hams in each workshop were randomly sampled, and three 3-cm-thick slices were taken from the central part of the Jinhua hams as described in Skrlep et al. were packed immediately and stored at −40 °C for further analysis (Skrlep et al. 2012).

### Volatile compounds analysis

Volatiles compounds analysis was carried out referring to the method of Lorenzo (Lorenzo 2014) and volatile compounds were extracted by solid phase micro-extraction technique (SPME) with a 10 mm long and 75 μm thick fiber coated with poly-dime-thylsiloxane. Twenty grams of minced sausages were used to extract volatiles compounds. Before the collection of volatiles, the fiber was preconditioned in the GC injection port at 250 °C for 20 min and then inserted into conical flask through the septum, afterwards exposed to the headspace for 40 min at 60 °C in a water bath. GC–MS analysis was performed with a GC–MS apparatus (Thermo Fisher Scientific, MA, USA). DB-5MS capillary column (30 m × 0.25 mm × 0.25 μm, J&W Scientific, Palo Alto, CA, USA) was used for the separation, the carrier gas was helium with the flow rate of 180 mL/min and the SPME fiber would be maintained at 250 °C. The temperature program was: from initial temperature 40 °C (1 min hold) to 130 °C at 5 °C/min, to 200 °C at 8 °C/min, to 250 °C at 12 °C/min and held for 7 min at 250 °C. The EI of mass spectrometer and GC–MS transfer line were all operated at 250 °C, detector voltage was 350 V, emission current was 150 μA, rate was 1 scan/s and *m/z* range was 33–500 for data collection. Compared with spectra from the NIST, the volatile compounds were identified and then quantified by calculating the ratio of individual compound peak area with the total peak area relatively.

### Pyrosequencing for 16S rDNA

According to the manufacturer’s instruction, the microbial DNA was extracted from the hams with PowerFood Microbial DNA Isolation kit (MO BIO Laboratories, Inc., USA). The V3 hypervariable region of the 16S rDNA was PCR amplified from the microbial genomic DNA using universal primer (forward primers: 5′-ACTCCTACGGGAGGCAGCAG-3′, reverse primers: 5′-TTACCGCGGCTGCTGGCAC-3′). PCR was subjected to 1 cycle of 98 °C for 5 min, followed by 25 cycles of denaturation at 98 °C for 30 s, annealing at 58 °C for 30 s and extension at 72 °C for 30 s, and finally extension at 72 °C for 5 min. Barcoded V3 amplicon was sequenced by Illumina Miseq at Personal Biotechnology Co., Ltd (Shanghai, China) using the pair-end method. All related sequence data have been deposited in the National Center for Biotechnology Information (NCBI) as a bio-project (BioProject ID: PRJNA354505, Accession Number: SRP093702). The samples were given the accessions numbers as SAMN 06046958 -SAMN06046966.

### Pyrosequencing data analysis

After pyrosequencing, all readings were screened and filtered using QIIME 1.6.0 software (Caporaso et al. 2010). Sequences reads with an average quality score lower than 25, ambiguous bases, homopolymer  > 7 bases, containing primer mismatches, or reads length shorter than 150 bp were removed. For V3 pair-end read, only sequences that overlapped more than 10 bp and without any mismatches were assembled. Operational taxonomic units (OTUs) were picked only if they had similar values of 97% or higher. Rarefaction curves and Venn plot were generated (Kõljalg et al. 2013). Alpha diversity was evaluated by community richness (Chao1 and ACE) (Pitta et al. 2010) and community diversity (Shannon and Simpson species) (Mahaffee and Kloepper 1997; Shannon 2001). All described analyses were performed using version 1.32.1 of MOTHUR software package (Schloss et al. 2009). Subsequent taxonomic affiliations were then obtained using the RDP classifier (http://rdp.cme.msu.edu/) with a confidence threshold cutoff of 0.8 to determine the taxonomy of the sequences (Wang et al. 2007). The abundances (percentages) were compared at the genus level (0.05 OTU) in each sample. The heat map were described using the statistical software package R (Ihaka and Gentleman 1996). The functional composition of communities were described using the statistical software PICRUSt (Langille et al. 2013) and annotated to their biological function according to KEGG (http://www.kegg.jp/kegg/pathway.html).

### Statistical analyses

All experiments in this study were repeated at least three times in independent experiments. Means and standard deviations were computed according to the experimental data. One way analysis of variance (ANOVA) with Tukey’s test was conducted on the data, and a *P* value at 0.05 was considered significant.

## Results

### Volatile flavor compounds in Jinhua ham

**Fig. 1:**
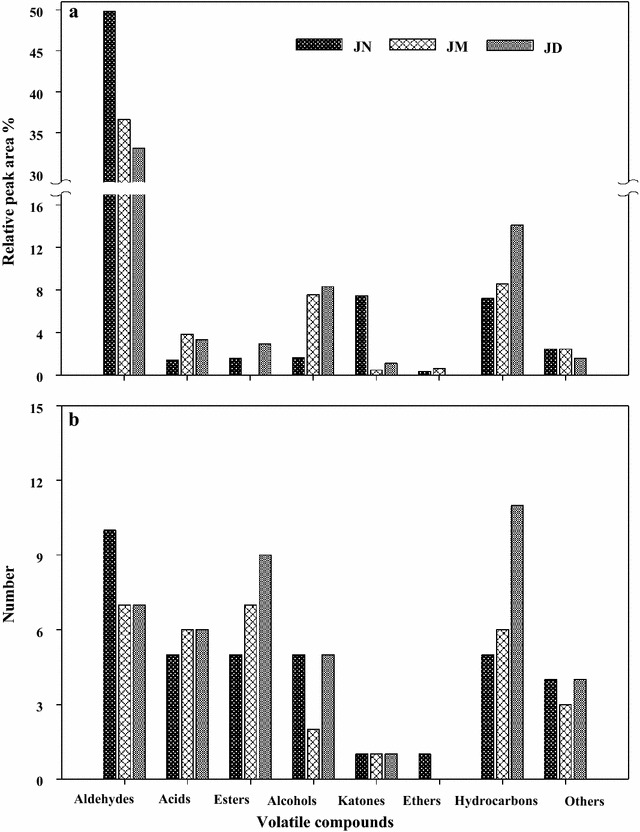
Volatile flavor compounds detected in Jinhua ham after post-ripening. **a** The relative peak area of volatile flavor compounds. **b** The number of volatile flavor compounds

To investigate the influences of long-term batch fermenting on the production of volatile flavor compounds, the volatile compositions in the Jinhua ham produced from the different aged workshops were detected using the GC–MS system (Fig. 1). Difference of total number of volatiles compounds and their relative contents among JN (30 years), JD (15 years) and JM (5 years) samples were observed. In this study, 72 volatile compounds that clustered in 8 chemical families were identified and quantified. Results indicated that the most abundant chemical family in flavor of Jinhua ham was aldehydes. After post-aging, the aldehydes contents in JN reached to 49.8% with 1.36- and 1.50-fold of JM and JD, respectively (Fig. 1). Similarly, based on relative peak area, ketones were the more abundant in the ham produced in JN, whereas acids, esters, ethers and hydrocarbons were more abundant in JM and JD. The results show that Jinhua hams have great differences in flavor among different workshop.

### Amplification and sequencing of Jinhua ham bacteria 16S rDNA gene sequences

The libraries containing the 16S rDNA gene sequences of bacteria targeting the V3 hypervariable region fragments of 16S rDNA gene were constructed. The entire pyrosequencing data set from the three samples contained 614,495 sequences. After filtering, 571,703 high-quality sequences (93.04% of the total sequences) remained with an average read length of 159 bp.

It is interesting that Chao1 and ACE in JN were higher than that in JD while lower than in JM (Table 1). Meanwhile, to estimate the overall diversity of ham bacteria, the Shannon and Simpson species richness index in each sample was also calculated (Table 1). The results showed that the ham bacteria were obviously different among the JN, JM and JD, suggesting that different years fermenting workshops had their own balanced microbial community structure and diversity.

**Table 1:** Alpha diversity in Jinhua ham

Sample	Reads	High-quality sequences	Abundance index		Diversity index	
			Chao1	ACE	Shannon	Simpson
JN	83,687	80,935	597.5	598.4	0.4491	4.5668
JM	84,282	80,776	827.8	844.4	0.7735	4.9827
JD	62,201	54,800	547.5	533.9	0.6512	3.2359

### Microbiota composition in ham markedly diversified in aged workshop

**Fig. 2:**
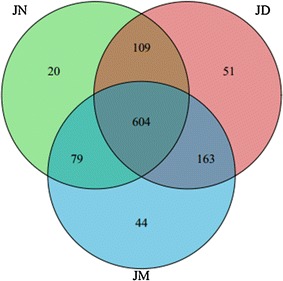
Venn plot for microbial diversity among ham produced from different workshop. The figures in different compartments mean the numbers of sequences specific for or common to ham workshop

**Fig. 3:**
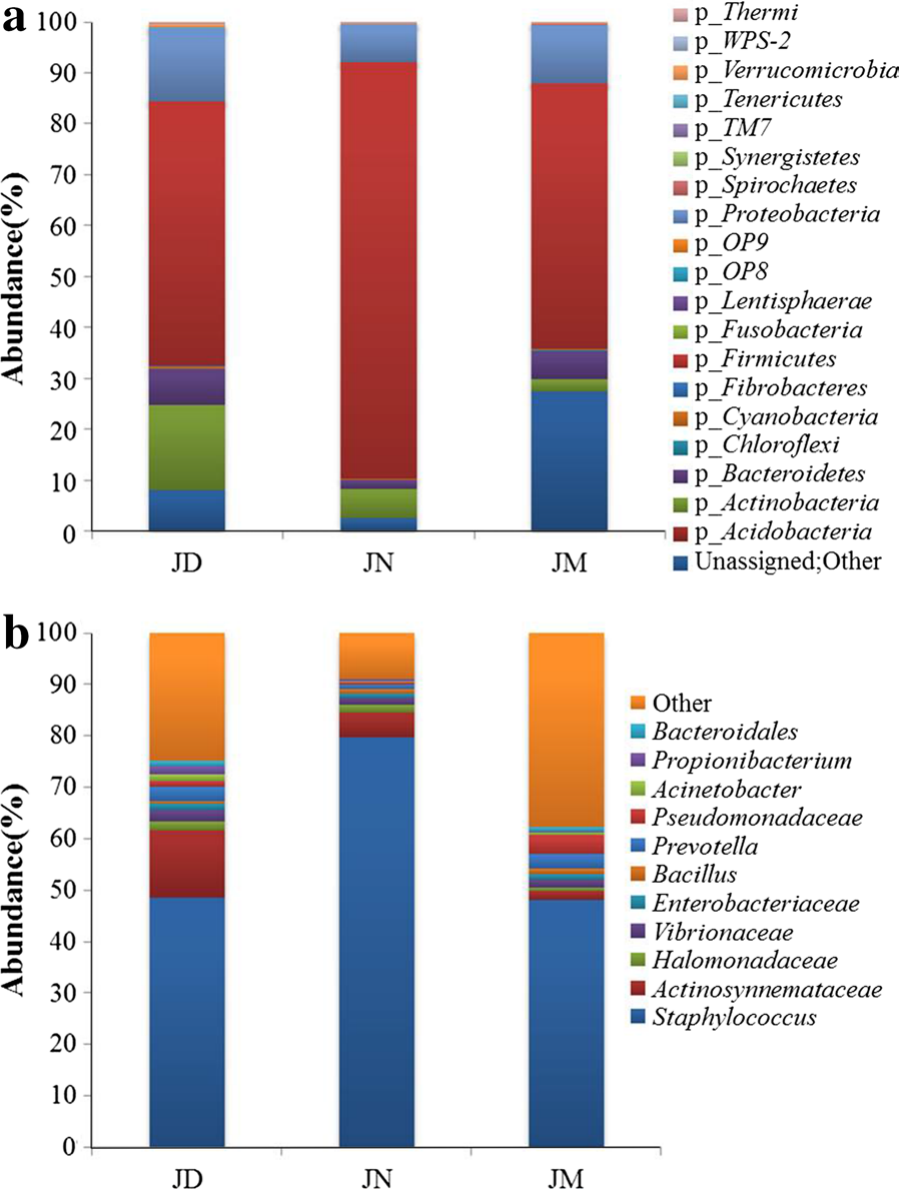
Changes in abundance of bacterial phyla based on 16S rDNA sequencing. **a** phyla-based; **b** family-based. In the legend, “k” stands for kingdom and “p” for phyla

**Fig. 4:**
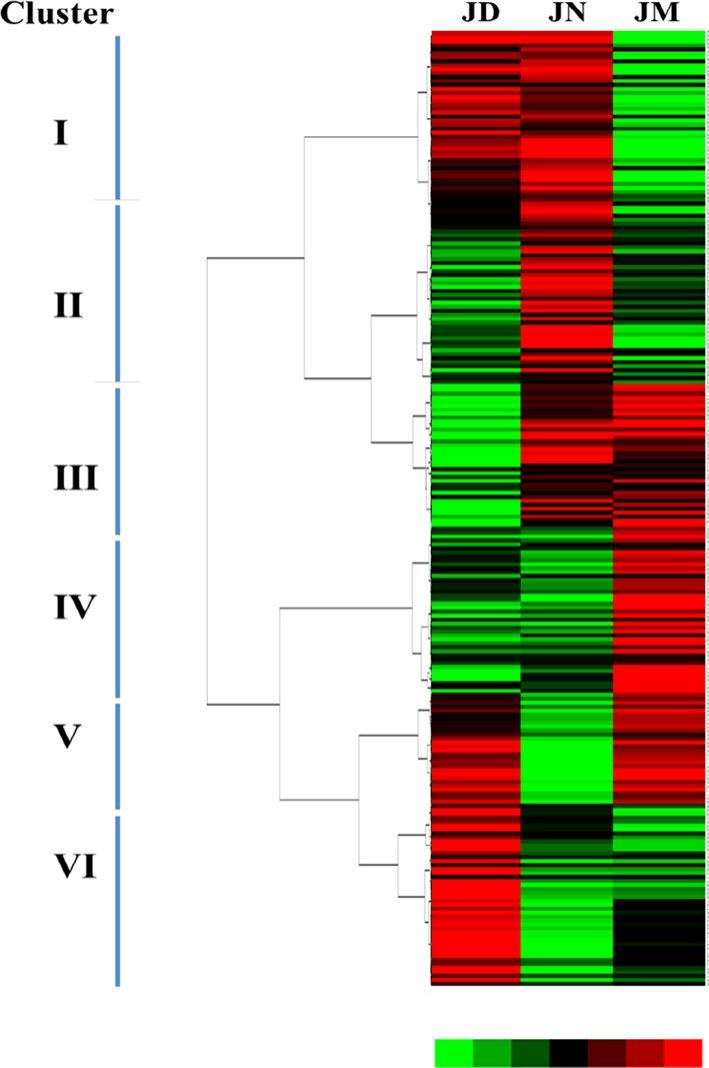
Heat map of the genera in JD, JN and JM ham. The heat map plot depicts the relative percentage of each genus (variables clustering on the* Y*-axis) within each sample (*X*-axis clustering). The values of genera based on the log2 transformed relative abundance were performed using the Gene Cluster 3.0 software. The results were visualized using the JAVA TREEVIEW software. The relative values for the genera are depicted by* color* intensity

The Venn plot indicated that 604 OTUs were common across all ham corresponding to some families of bacteria existing among three hams (Fig. 2). The microbiota in Jinhua ham was constituted of nineteen phyla and the vast majority of sequences belong to one of the four major phylas: *Bacteroidetes*, *Actinobacteria*, *Proteobacteria* or *Firmicutes* (Fig. 3a). The abundance of the remaining phyla was less than 1.5% of total sequences including *Acidobacteria*, *Chloroflexi*, *Cyanobacteria*, *Fibrobacteres*, *Fusobacteria*, *Lentisphaerae*, *OP8*, *OP9*, *Spirochaetes*, *Synergistetes*, *TM7*, *Tenericutes*, *WPS*-*2* and *Thermi*. Among all hams, *Firmicutes* was the most predominant microbiota and its abundance was more than 52.00% even as high as 81.80% in JN. However, the abundance of *Bacteroidetes* and *Proteobacteria* in JN both lower than that in JD and JM. Additionally, The total bacterial sequences from the three ham samples located in 242 genera. These bacterial genera and their abundances in the JD, JN and JM ham were shown in the heat map (Fig. 4). The map presents that the bacteria in ham were markedly different in composition form. The clustering analysis led to the division of the 242 genera into 6 prominent categories (Fig. 4). The intensity of genera belonging to clusters I in JD and JN was higher than that in JM. Those genera in clusters II abounded in JN ham and their abundance in JD and JM was relatively lower. It was noted that the abundance of genera belonging to clusters III, IV and V was higher in JM, but they showed significant diversity in JD and JN. Differences among the genera in clusters VI also presented in three ham samples. To observe the changes in the ham closely, the 11 most abundant genera that appeared in all three samples was compared. The results showed that after a decade of fermentation acclimation, the distribution differences of bacteria were reduced (Fig. 3b). For example, among all hams, the most frequently detected genus was *Staphylococcus* which changed in different ham samples. This genus accounted for 79.71% of the total in JN, but decreased markedly in JD (48.58%) and JM (48.16%). *Actinosynnemataceae* sensu stricto ranked the second only to *Staphylococcus* in JD, but the genus was less in JM. It is also noted that there were many bacterial sequences whose taxonomic status could not be defined. Furthermore, some sequences could not be defined of their taxonomic status, even at the phylum level. The changes of these unclassified bacteria became greatly among different hams and its composition markedly increased in JM.

### Functional composition diversity varying with age of workshops

**Fig. 5:**
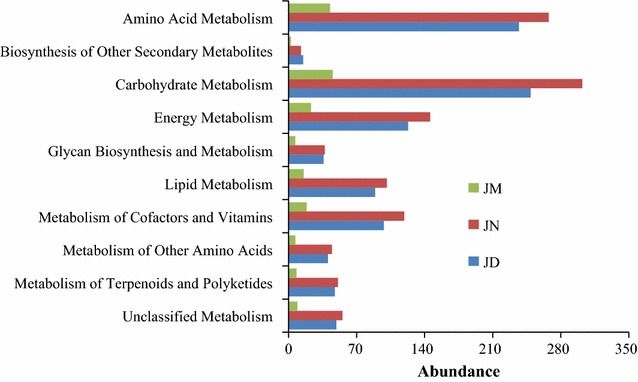
Functional genes related to metabolism in Jinhua ham. The functions of OTUs were assigned according to KEGG

Predicted functional class analysis showed that these OTUs were divided into 7 functional classes including cellular processes, environmental information processing, genetic information processing, human diseases, metabolism, organismal system and unclassified. In the present study, in order to effectively analyze the difference in the ham, OTUs involved in metabolism that appeared in Jinhua hams was compared and it showed their own different metabolic process (Fig. 5). The abundance of amino acid metabolism-related and carbohydrate metabolism-related OTUs were significantly higher than other metabolism-related. Specifically, the entire metabolism-related OTUs in different hams had sharp distinction indicating that the different years fermenting benefits the Jinhua ham flavor formation for their difference of complex metabolism, which depends on their own well-balanced bacteria community.

## Discussion

The workshop microbiota is recognized to play a role in the flavors of Jinhua ham. In the present study, microbial community structure and diversity of different ages of ham workshops were investigated by the high-throughput pyrosequencing technique. Our results provide comprehensive understanding of microbial community structure and diversity among different aged workshops which produced special flavor Jinhua hams.

### Difference in Workshop lead to entirely different sets of microbial community structure

Microorganisms in ham are involved in the fermentation process to produce the aromatics which determine the ham flavor style which were routinely selected by the fermentation process. The genera *Staphylococcus* (Cordero and Zumalacarregui 2000; Fulladosa et al. 2010) were identified from dry-cured ham samples by cultured or un-cultured methods in previous studies. Here, we identified 242 genera as the core microbiota in Jinhua ham samples using the pyrosequencing technique. Consistent with previous reports, despite the shifted percentage in different ham, the most frequently detected genus also was *Staphylococcus* among all hams in the present study. Additionally, the present study indicated that microbial communities in the JM (5-year workshop) were different from those in the JD (15-year) and JN (30-year). The diversity of prokaryotes decreased with workshop age and sustained advantage and balance in the JN (Fig. 4), while those in the JD were in the transition state. According to our results, three distinct phases were separated by the changes of workshop microbial communities. Phase A was high in diversity and species richness which was possibly resulted from biogeochemical environment similar to the surrounding air environment. At this phase, the abundance of genera belonging to clusters III, IV and V was higher (Fig. 4). Additionally, there were so many unassigned bacteria in phase A but decreased in phase B (Fig. 3). The community structure of Phase B dramatically changed and significant decreased in prokaryotic diversity. This could be due to the fact that the microbial community was optimized and adapted at very different environmental conditions (e.g., temperature and humidity) created by the Jinhua ham process in the workshop. However, the abundance of *Actinobacteria* and *Proteobacteria* increased (Fig. 3). The abundance of genera belonging to clusters I in Phase B was consistent with Phase C. Phase C was the relative mature period of the ham microbial community. Microbial diversity was stable in this phase and was significantly lower than in the young workshop. The specific microbial distribution in the ham may be resulted in periodic fermentation and enrichment for more than 30 years without interruption. Moreover, mutual collaborations and interactions among different bacteria species could lead to a well-balanced bacteria community in the biogeochemical environment resulting in ham flavor different.

### Dominant bacteria communities and their relationships to Jinhua ham flavors

Bacteria communities play a crucial role in the production of flavor of the dry-cured meat product (Fadda et al. 2010; Kaban 2013). Volatile compounds are generated from the catabolism of proteins, lipids, and carbohydrates through the action of microbial and endogenous enzymes during process due to a high natural microbiota background (Lücke 1996; Zeuthen 2007). The total number of volatile compounds identified in the present study and/or their relative contents were different. Studies have shown the dominant volatile compounds in Bayonne, Jinhua, Corsican, Iberian, Parma and Serrano dry-cured hams were aldehydes (Huan et al. 2005; Mottram 1998). It is not surprising that the content of aldehydes increased over age of the workshop which had well-balanced bacteria community in the present study. Moreover, hexanal with an odor of green leaves is the dominated profile of aldehydes and is synthesized during the oxidation of unsaturated fatty acids. Hexanal was found at the highest level in JN while lowest in JM. Previous works have been carried out to study the changes in volatile aldehydes and ketones in dry-cured ham. Those results showed that flavor formation by secondary metabolism of microorganisms, especially amino acid catabolism in which methyl-branched aldehydes and methyl ketones were generated (Hinrichsen and Andersen 1994; Narváez-Rivas et al. 2014). Additionally, since OTUs involved in metabolism of terpenoids and polyketides, amino acids, lipid and carbohydrate in JN differed from in JM and its flavor could have their own typical characteristics. In conclusions, the microbial diversity and community structure markedly changed in different aged workshops which corresponded to the strong flavoring characteristics of Jinhua ham.

In conclusion, GC–MS assays indicated that remarkably difference among Jinhua hams concerning number of volatiles compounds and their relative contents. Hams produced in the older workshop contained the higher concentrations of aldehydes that are important for the ham flavor. Meanwhile, bacterial diversity and microbial community structure were significantly different among three factories. Three-phase model to characterize the changes of ham microbial communities was demonstrated. Importantly, these results indicated that aged factories could accumulate adaptive microbes, and well-balanced microbial diversity was responsible for the production of more flavorful Jinhua ham.

## Abbreviations

DNA: deoxyribonucleic acid
GC–MS: gas chromatography–mass spectrometry
SPME: solid phase micro-extraction technique
OTUs: operational taxonomic units
NIST: National Institute of Standards and Technology
PCR: polymerase chain reaction
NCBI: National Center for Biotechnology Information
